# Evaluation of genetic alterations in hereditary cancer susceptibility genes in the Ashkenazi Jewish women community of Mexico

**DOI:** 10.3389/fgene.2023.1094260

**Published:** 2023-02-10

**Authors:** Clara Estela Díaz-Velásquez, Rina Gitler, Adriana Antoniano, Ronny Kershenovich Sefchovich, Aldo Hugo De La Cruz-Montoya, Héctor Martínez-Gregorio, Ernesto Arturo Rojas-Jiménez, Ricardo Cortez Cardoso Penha, Luis Ignacio Terrazas, Talia Wegman-Ostrosky, Ephrat Levi-Lahad, Jovanny Zabaleta, Sandra Perdomo, Felipe Vaca-Paniagua

**Affiliations:** ^1^ Laboratorio Nacional en Salud, Diagnóstico Molecular y Efecto Ambiental en Enfermedades Crónico-Degenerativas, Facultad de Estudios Superiores Iztacala, Tlalnepantla, Estado DeMéxico, Mexico; ^2^ Fundación Alma, Mexico City, Mexico; ^3^ Instituto Nacional de Medicina Genómica, Mexico City, Mexico; ^4^ Unidad de Biomedicina, Facultad de Estudios Superiores Iztacala, UNAM, Tlalnepantla, Estado DeMéxico, Mexico; ^5^ Genomic Epidemiology Branch, International Agency for Research on Cancer (IARC/WHO), Lyon, France; ^6^ Subdirection of Basic Research, Instituto Nacional de Cancerología, Mexico City, Mexico; ^7^ Department of Medical Genetics, Shaare Zedek Medical Center, Jerusalem, Israel; ^8^ Departament of Interdisciplinary Oncology, School of Medicine, LSU Health New Orleans, New Orleans, LA, United States; ^9^ Stanley S. Scott Cancer Center, LSU Health New Orleans, New Orleans, LA, United States

**Keywords:** Ashkenazi Jews, genetic screening, panel of genes, massive parallel sequencing, founder variant, APC

## Abstract

**Background:** Individuals of Ashkenazi Jewish ancestry have been identified as having higher prevalence of specific pathogenic variants associated with susceptibility to specific rare and chronic diseases. In Mexico, the prevalence and composition of rare cancer predisposing germline variants in Ashkenazi Jewish individuals has not been evaluated.

**Aim and methods:** We aimed to evaluate the prevalence of pathogenic variants by massive parallel sequencing in a panel of 143 cancer-predisposing genes in 341 women from the Ashkenazi Jewish community of Mexico, who were contacted and invited to participate in the study through the ALMA Foundation for Cancer Reconstruction. Pre- and posttest genetic counseling was given and a questionnaire on personal, gyneco-obstetric, demographic and lifestyle variables was conducted. From peripheral blood DNA, the complete coding region, and splicing sites of a panel of 143 cancer susceptibility genes, including 21 clinically relevant genes, were sequenced. The Mexican founder mutation *BRCA1 ex9-12del* [NC_000017.10(NM_007294):c. (825+1–826-1)_(4,589+1–4,590-1)del] was also evaluated.

**Results:** Among study participants (mean age ±standard deviation: 47 ± 14) 15% reported a personal history of cancer (50/341). Fourteen percent of participants (48/341) were carriers of pathogenic and likely pathogenic variants distributed among seven high-risk genes (*APC, CHEK2, MSH2, BMPR1A, MEN1, MLH1,* and *MSH6*), whereas 18.2% (62/341) had variants of uncertain clinical significance in genes associated with breast and ovarian cancer susceptibility (list of genes with VUS). Pathogenic and likely pathogenic variants in 16 susceptibility genes with ambiguous or non-well-established risk association for cancer were detected in 17.6% (60/341) of participants. Sixty four percent of participants reported current alcohol consumption compared with the 39 percent prevalence of alcohol consumption in Mexican women. None of the participants carried the recurrent Ashkenazi and Mexican founder mutations in *BRCA1* or *BRCA2*, but 2% (7/341) had pathogenic Ashkenazi Jewish founder variants in *BLM*.

**Conclusion:** Our findings show a diverse pathogenic variant composition among the recruited individuals of Ashkenazi Jewish ancestry in Mexico consistent with being a high-risk population for genetic diseases, which warrants further investigation to adequately assess the burden of hereditary breast cancer in this group and implement appropriate preventative programs.

## Introduction

The Ashkenazi Jewish (AJ) community has been identified as a group with elevated risk for numerous genetic diseases including hematological, biochemical diseases, and various types of cancers, most likely because of a genetic bottleneck that preceded a period of rapid population growth (Behar et al., 2004). This historical trajectory fostered a high prevalence of genetic variants related with numerous dominant and recessive conditions (Gusev et al., 2012; Palamara et al., 2012). The Jewish community represents the third religious group in the Mexican population (INEGI, 2020) and constitutes the third largest Jewish community in Latin America (Avni et al., 2011).

In Mexico, breast cancer is the leading cause of cancer-related deaths among women aged 20 to 59, and 5%–10% of cases can be explained by hereditary genetic factors. Pathogenic and Likely Pathogenic (P/LP) genetic variants in the *BRCA1/2* (Breast Cancer 1/2) genes are the main cause of hereditary breast cancer. Among individuals of AJ ancestry, most breast cancers are due to three *BRCA1/2* founder mutations (*BRCA1*: 185delAG [c.68_69del], 5382insC [c.5266dup]; *BRCA2*: 6174delT [c.5946del]); while in the Mexican population the founder mutation *BRCA1* ex9-12del has a high frequency. The prevalence of pathogenic variants in non-*BRCA* genes has not been widely investigated in the Latin American Region generally, or Mexico specifically (Cock-Rada et al., 2018; Quezada Urban et al., 2018; Adaniel et al., 2019; Oliver et al., 2019; Sandoval et al., 2021; Solano et al., 2021).

While various studies have identified germline pathogenic variants associated with hereditary cancer syndromes in the Mexican population, there is currently no information on genetic alterations specifically focused on Mexicans who self-identify as AJ (Torres-Mejía et al., 2015; Villarreal-Garza et al., 2015; Moreno-Ortiz et al., 2016; Quezada Urban et al., 2018; Gallardo-Rincón et al., 2020). The lack of this information remains an important limitation to further evaluate the implications of identifying high risk populations in genomic testing and public health policies in Mexico. To address this gap, we conducted a genetic analysis of 143 genes associated with a variety of cancers to ascertain the prevalence and composition of pathogenic genetic variants among individuals of AJ ancestry who were born and live in Mexico.

## Materials and methods

### Study population and data collection

Women were contacted and invited to participate in the study in community centers through the ALMA Foundation for Cancer Reconstruction (https://www.alma.org.mx/), a recognized charity organization within the AJ community in Mexico City. Inclusion criteria for recruitment included: all women self-identified as AJ and who have an AJ mother or father, as well as AJ maternal or paternal grandparents. Adopted women were excluded.

The protocol was approved by the Ethics Committee of the Faculty of Health Sciences of the Anahuac University (201923) and was conducted in accordance with the Declaration of Helsinki. A total of 341 women over the age of 18, of Mexican nationality accepted to participate in the study. All participants provided written informed consent for participation after a detailed explanation of the study, and their samples were anonymized and sent to the Laboratorio Nacional en Salud: Diagnóstico Molecular y Efecto Ambiental en Enfermedades Crónico-Degenerativas, Facultad de Estudios Superiores Iztacala, UNAM. All participants had pre-test and post-test genetic counseling provided by two clinical geneticists.

### Sample preparation and DNA extraction

Four mL of whole blood was drawn from each participant and stored at −80°C locally. The time interval between collection and freezing of samples was never greater than 36 h. Peripheral blood DNA was extracted according to the manufacturer’s instructions using the DNeasy Blood & Tissue Kit (Qiagen). The DNA concentration was determined using the Invitrogen Qubit dsDNA HS Assay Kit, and the material’s integrity and purity were determined using agarose gel electrophoresis and spectrophotometry.

### Epidemiological information on additional risk factors

All participants were invited to complete a lifestyle questionnaire including information on socioeconomic status, health, and reproductive history (age at menarche, pregnancy, number of births, age at each birth, breastfeeding), use of hormones (e.g., oral contraceptives), smoking and alcohol habits, maximum attained weight, and body mass index (BMI), and personal history of cancer. Information on family history of cancer was limited without precise details on degree of siblings, family arm or cancer types reported, and therefore was excluded from the analyses. Information was collected using REDCap electronic data capture tools (Harris et al., 2009; Harris et al., 2019).

### Statistical analysis

Age and BMI were included as continuous variables, whereas the rest of the factors were considered as categorical variables. Differences in clinicopathological characteristics between individuals with and without pathogenic variants were analyzed using *X2* test and a *p*-value <0.05 was considered statistically significant. All the statistical analyses were performed using R (www.r-project.org).

### Library preparation and sequencing

Library preparation was performed as described previously (Quezada Urban et al., 2018). Briefly, The GeneRead Cancer Predisposition V2 Kit (Qiagen) was used to prepare the library, which targets 143 genes whose loss of function is a well-known mechanism associated with more than 80 inherited oncologic diseases based on data from the College of American Pathologists (CAP) guidelines, the National Comprehensive Cancer Network (NCCN) guidelines, and The Cancer Genome Atlas (TCGA). The amplification was carried out in four-pool PCR reactions and all libraries were barcoded and diluted equimolarly. Sequencing was performed in a HiSeq 4000 (Illumina) with pair-end (2x150) chemistry and to a theoretical coverage of 2000X.

### Bioinformatic analyses and variant calling

Bioinformatic analyses were performed as previously described (Quezada Urban et al., 2018). Briefly, BWA (Li and Durbin, 2009) and GATK were used to perform alignment and variant calling (Van der Auwera et al., 2013). With BWA-MEM, fastq files were aligned to the human genome reference hg19; indels were realigned and bases were recalculated. Adaptors were soft-clipped and reads with a length of less than 20 bp were discarded. HaplotypeCaller was used to determine genetic variants (Van der Auwera et al., 2013). ANNOVAR and InterVar were used to annotate variants (Wang et al., 2010; Li and Wang, 2017). The Human Genome Variation Society (HGVS) nomenclature was used to describe the genetic variants (den Dunnen et al., 2016). The classification of variants followed the American College of Medical Genetics and Genomics’ (ACMG) five-tier criteria (Richards et al., 2015) and was manually curated. We excluded synonymous variants, those with a depth of <10X or a mutant allele fraction of <15%, those found in homopolymeric tracts (>8 bp) or spurious variants with conflicting patterns, including those in only one strand, within erroneous base tracts or at the end of only one of the amplicons (with no redundancy in additional amplicons). All splicing and null variants (stop-gain/loss, frameshift indels), as well as missense variants, were considered pathogenic (P) or likely pathogenic (LP) as defined by the ACMG guidelines and if reported as pathogenic in ClinVar (ACMG supporting evidence of pathogenicity PPP5) (Landrum et al., 2020). In addition, we used VarSome and InterVar to compare the ClinVar information and determine more precisely the variant classification. Null variants located at downstream than 50 bp of the final splice junction at the 3′extreme end of the gene were excluded. Minor allelic frequency 0.001 in non-Ashkenazi populations was used as a threshold to eliminate common human variation with the gnomAD, ExAC and the 1,000 Genomes (1000 G) project databases. Variants with higher Ashkenazi Jew specific population frequency were evaluated manually to account for bottleneck population effects in this population. All filtered variants were manually curated using the IGV software (Thorvaldsdóttir et al., 2013). The Leiden Open Variation Database (Fokkema et al., 2021) was also used to investigate variants in *MLH1, MSH2, MSH6*, and *PMS2*. Ninety six percent of the pathogenic and likely pathogenic variants in high-risk genes were validated by Sanger sequencing.

### Detection of the Mexican founder mutation in *BRCA1*

PCR based sequencing limits the identification of large deletions including the highly prevalent Mexican founder mutation *BRCA1 ex9-12del* [NC_000017.10(NM_007294):c. (825+1-826-1)_(4,589+1–4,590-1)del]*.* Therefore, this pathogenic variant was evaluated by PCR amplification of the mutant and wildtype allele, using specific primers based on the method developed by Weitzel et al. (2007), with a positive control previously validated by multiplex ligation-dependent probe amplification (MLPA). PCR products were resolved in 1.5% agarose gels to identify the amplification of the truncated allele and sequenced.

### Genetic ancestry component

To evaluate the ancestral genetic composition of the study participants DNA samples were genotyped at the University of Minnesota Genomics Center with the MassARRAY System (Agena Bioscience) with a panel of 104 ancestry informative markers (AIMs) that can be used to estimate Indigenous American, African, and European ancestry as previously shown (Fejerman et al., 2008; Serrano-Gomez et al., 2016). Single nucleotide polymorphisms with call rate <90% (1 polymorphism) or that deviated from Hardy–Weinberg equilibrium were removed from the analysis. We used PLINK with subsequent principal component analysis (PCA) to detect ancestral structure using 1000vG as reference population as an initial quality control step (Supplementary Table S1) (Tang et al., 2005). A total of 341 cases were genotyped, but only 327 cases remained after excluding samples with genotype call rates <90%. The final set available for analysis included 327 participants and 103 AIMs. We used a maximum likelihood (ML) approach for ancestry estimation and included available reference data from the 1000 G project. Genotype data were converted into PLINK pgen format using PLINKv.2.0 (Chang et al., 2015).

Of the 103 AIMs, 58 were found in the 1,000 Genomes dataset (Auton et al., 2015). To detect and remove variants in linkage disequilibrium, an independent pairwise test (LD window size of 50 kb and r2 threshold of 0.2) was performed using PLINK, remaining 57 independent variants that were used in the genetic ancestry analyses. The genotype data with 327 cases was then merged with the 1,000 Genomes genotype data for the reference populations (Europeans: British, Finnish, Iberian, Northern and Western European, and Toscani; Latin-Americans: Mexican, Peruvian, Colombian and, Puerto Rican; African:Yoruba).

## Results

### Demographic characteristics and risk factors of Mexican Ashkenazi Jewish

The demographic profile of participants was characterized by a normal BMI, a high level of education (university and postgraduate: 91%), high maternity rate and age of first pregnancy before the age of 30. More than 50% of participants were never smokers but current drinkers (Table 1). Fifteen percent (50/341) of individuals had a personal history of cancer, including 10 women previously diagnosed with breast cancer, 3 with ovarian cancer, 2 with colorectal cancer and 1 with pancreatic cancer.

**TABLE 1:** Demographic characteristics of women of Ashkenazi Jewish ancestry who reside in Mexico City (*N* = 341).

Characteristic	Mean (SD), N (%), median (IQR)
Age	47 (14)
Current BMI (Kg/m2)	23.0 (21.0, 26.3)
20 y BMI (Kg/m2)	20.8 (19.1, 22.8)
Educational level	
Illiterate	0 (0%)
Primary	0 (0%)
Secondary	3 (0.9%)
Superior/Technical	28 (8.2%)
University	179 (53%)
Postgraduate	130 (38%)
No data available	1
Personal history of cancer	
yes	50 (15%)
No	289 (85%)
Not known	2 (0.6%)
Parous	289 (85%)
Age at first full term pregnancy**	26 (24, 28)
No data available	55
Contraceptive use***	301 (88%)
Months breastfeeding**	6 (2, 8)
No data available	52
Tobacco consumption^a^	
Never	220 (65%)
Former	79 (23%)
Current	42 (12%)
Alcohol consumption^a^	
Current	218 (64%)
Former	6 (1.8%)
Never	117 (34%)

^a^ For the Tobacco/Alcohol consumption variables, current smokers and current drinkers included those women who reported daily smoking or drinking at time of recruitment. **Pregnancy variable included women who had at least one full term pregnancy. ***Contraceptive use refers to consecutively use during the last year.

### Genetic ancestry composition

**FIGURE 1:**
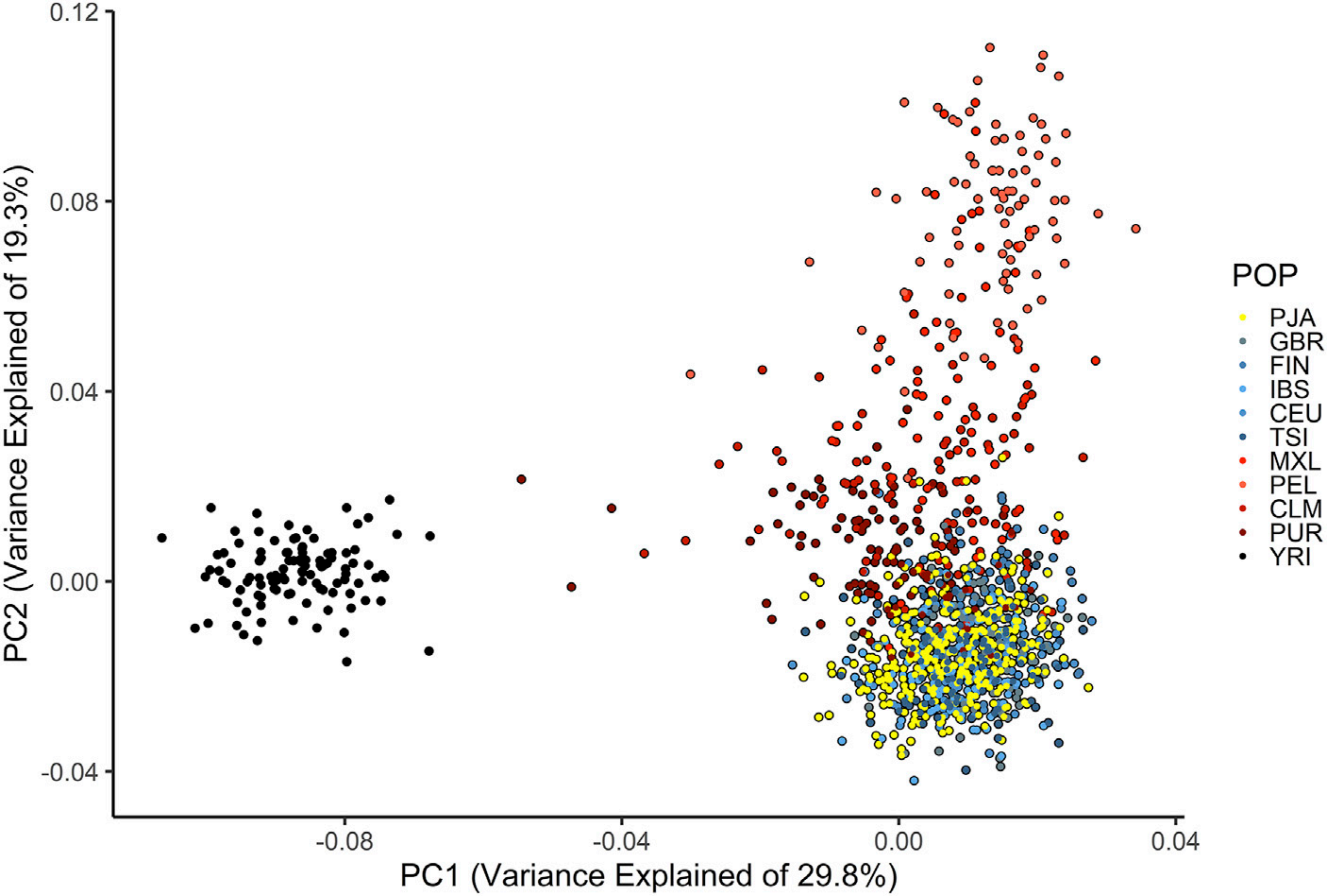
Principal component analysis showing ancestral composition of the AJ women recruited in comparison with the reference populations from 1000 G. PJA, Population of Mexican Ashkenazi Jews represented by yellow dots. POP, Populations, GBR, British, FIN, Finnish, IBS, Iberian, CEU, Northern and Western European, TSI, Toscani, MXL, Mexican, PEL, Peruvian, CLM, Colombian, PUR, Puerto Rican, YRI, Yoruba.

Ancestry categories based on self-identity are more comprehensive because they account for individual knowledge of ancestry similarities including cultural and social components. In addition, we identified the genetic ancestry fractions of the participants using the allelic composition of 103 ancestry informative SNPs. We used principal component analysis which showed that 92.3% (302/327) of the individuals genotyped had more than 70% allelic composition matching those in the reference populations grouped in Europe (Figure 1; Supplementary Table S1). The allelic frequencies of those 25 individuals below the 70% cutoff ranged between 56%–69% and grouped with the reference Latin American populations from the 1000 G (CLM, MLX, PUR) (Figure 1).

### High-risk pathogenic variants detected

#### Pathogenic and likely pathogenic variants detected in high and moderate risk genes

**FIGURE 2:**
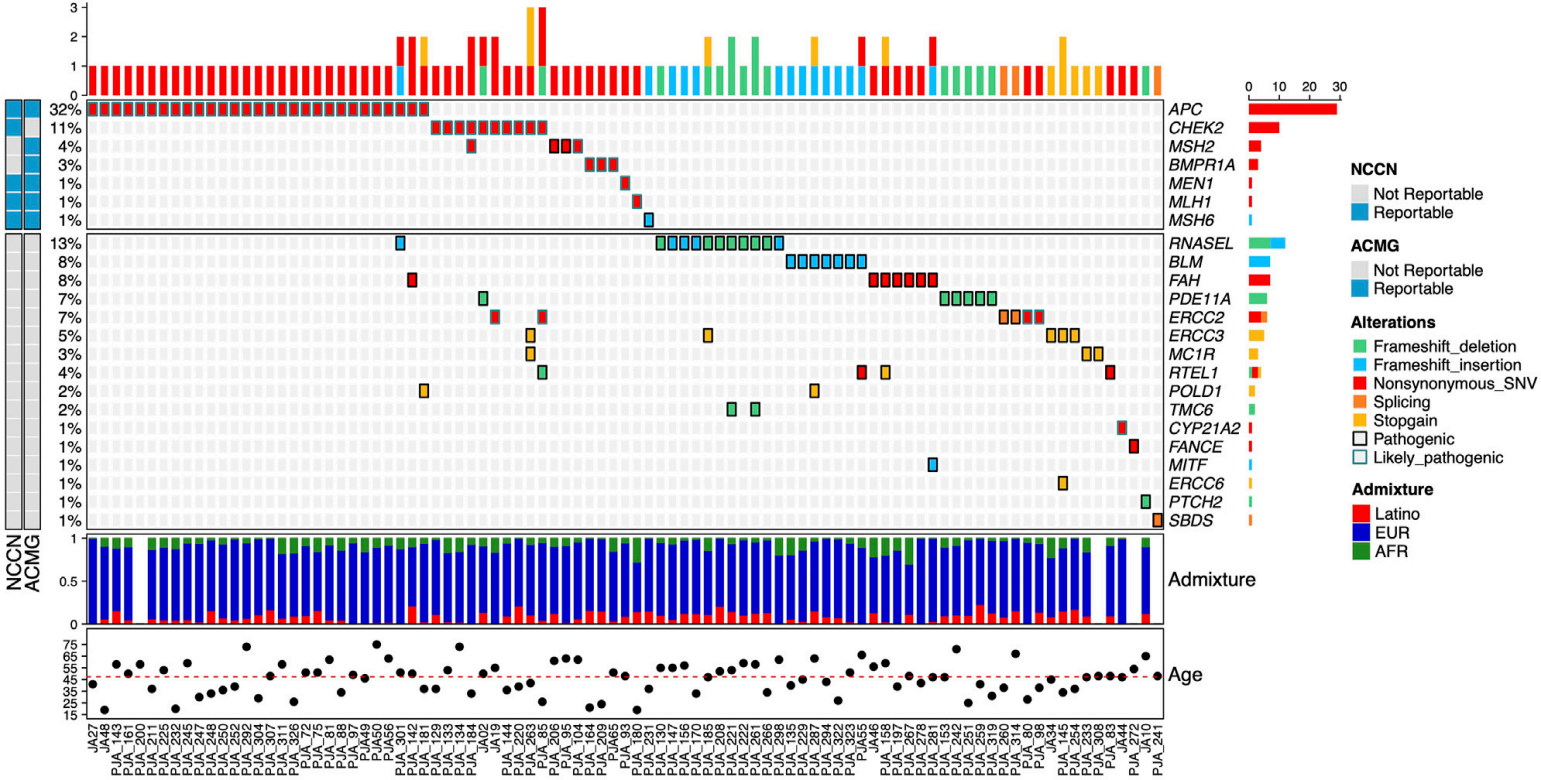
Allelic distribution of pathogenic variants in high-risk genes for HBOC and genes with unknown risk level. The relative frequencies, type of mutation, and NCCN and ACMG reportable genes are shown in the upper panel. The center panel illustrates pathogenic variants detected in genes with unknown or insufficiently documented risk of HBOC. The lower panel represents the three-component admixture distribution of the participants. The mutation type (alterations) and admixture component are color-coded. The relative frequency and total count for gene alterations are shown in the left and right.

Overall, we identified 58 pathogenic (P) and 51 likely pathogenic (LP) variants in 26.7% of the individuals (91/341), distributed across 23 genes (Figure 2). Nevertheless, only 14% had P/LP variants in clinically relevant genes (48/341). Seven high-risk genes considered reportable by the ACMG SF v3.0 (Miller et al., 2021) and the NCCN Version 2.2021 guidelines for Breast, Ovarian and Colorectal cancers (Benson et al., 2021; Daly et al., 2021) were affected and included *APC* (8.5%; 29/341), *CHEK2* (2.9%; 10/341), *MSH2* (1.2%, 4/341), *BMPR1A* (0.9%; 3/341), *MEN1* (0.3%; 1/341), *MLH1* (0.3%; 1/341), and *MSH6* (0.3%). We detected 10 pathogenic alleles in these seven genes (Table 2).

**TABLE 2:** Pathogenic and likely pathogenic alterations in high-risk genes detected in 341 participants.

Frequency (n, %)	Gene	Type of change	Transcript	Exon	cDNA change	Protein change	Zygosity	Variant interpretation
26 (7.62)	*APC*	Non-syn	NM_000038	16	c.3920 T>A	*p*.Ile1307Lys	Heterozygous	LP
1 (0.29)	*APC*	Non-syn	NM_000038	16	c.3920 T>A	*p*.Ile1307Lys	Homozygous	LP
2 (0.58)	*APC*	Non-syn	NM_000038	9	c.932 A>G	*p*.Lys311Arg	Heterozygous	LP
4 (1.17)	*CHEK2*	Non-syn	NM_007194	12	c.1270 T>C	*p*.Tyr424His	Heterozygous	LP
6 (1.75)	*CHEK2*	Non-syn	NM_007194	12	c.1283C>T	*p*.Ser428Phe	Heterozygous	LP
2 (0.58)	*MSH2*	Non-syn	NM_000251	13	c.2086C>T	*p*.Pro696Ser	Heterozygous	*p*
2 (0.58)	*MSH2*	Non-syn	NM_000251	6	c.1045C>G	*p*.Pro349Ala	Heterozygous	LP
3 (0.87)	*BMPR1A*	Non-syn	NM_004329	9	c.676G>T	*p*.Val226Phe	Heterozygous	LP
1 (0.29)	*MEN1*	Non-syn	NM_000244	9	c.1343C>A	*p*.Ser448Tyr	Heterozygous	LP
1 (0.29)	*MLH1*	Non-syn	NM_000249	11	c.976G>A	*p*.Val326Met	Heterozygous	LP
1 (0.29)	*MSH6*	FI	NM_000179	9	c.3980_3981insTCAG	*p*.Leu1330Valfs*12	Heterozygous	*p*

Non-syn, non-synonymous variant, FI, frameshift insertion, *p*, pathogenic, LP, likely pathogenic.

Eight participants had more than one P or LP variant: seven participants had one LP variant in a high-risk gene (*APC* and *CHEK2*) and up to two additional *p* or LP variants in genes of unknown risk (*RNASEL*, *FAH*, *PDE11A*, *ERCC2*, *ERCC3*, *MC1R*, *RTEL1*, and *POLD1*). One participant had two LP variants in *CHEK2* and *MSH2* (Figure 2).

The P variant *APC p*.Lys311Arg initially classified as a VUS, was reclassified as LP based on the participants’ family history of adenomatous polyposis. This variant was present in two consanguineous members of the same family. Presence of P/LP variants was significantly associated with age of participants at recruitment (*p* = 0.043). We did not find significant associations between lifestyle factors variables (BMI, alcohol consumption, reproductive factors) or personal history of cancer and the presence of P or LP variants (Supplementary Table S2).

#### Pathogenic variants associated with hereditary breast and ovarian cancer (HBOC) syndrome

In comparison to other populations, the AJ community has a higher prevalence of founder mutations associated with HBOC. None of the participants analyzed had the recurrent Ashkenazi founder mutations in *BRCA1* or *BRCA2* (Levy-Lahad et al., 1997). We also tested participants for the high-frequency *BRCA1* ex9-12del*.* Mexican native founder variant without any positive result. Other HBOC P and LP variants in moderate penetrance genes were found in 13 participants and were *CHEK2 p*.Ser428Phe and *p*.Tyr424His, detected in six and four participants, respectively and the variants *p*.Pro696Ser and *p*.Pro349Ala in *MSH2*, found in two participants. None of the participants harboring these variants had personal history of cancers associated with HBOC syndrome.

#### Variants of unknown clinical significance (VUS) associated with hereditary breast and ovarian cancer (HBOC) syndrome

In total, 18.2% (62/341) of the participants harbored monoallelic variants of uncertain significance (VUS) in genes of clinical relevance for HBOC (Supplementary Table S3). These variants were detected in *BRIP1* (2.3%; 8/341), *ATM* (2%; 7/341), *MSH6* (1.8%; 6/341), *FANCI* (1.5%; 5/341), *KIT* (1.2%; 4/341), *RET* (1.2%), *MSH2* (0.9%; 3/341), *BRCA2* (0.9%), *BARD1* (0.9%), *PALB2* (0.9%), *PMS2* (0.9%), *ERCC2* (0.9%), *CHEK2* (0.6%; 2/341), *FAH* (0.6%), *NF1* (0.3%; 1/341), *NF2* (0.3%), *BRCA1* (0.3%), *MEN1* (0.3%), *PDE11A* (0.3%), and *RAD51C* (0.3%).

#### Pathogenic and likely pathogenic variants in genes of uncertain cancer risk

We detected 50 participants with 20 unique *p* and LP variants distributed in 16 susceptibility genes with uncertain or non-well-established risk association for cancer (Table 3) but only nine of these participants had a personal history of cancer. Ten participants had more than one P or LP variant, of which nine had two P variants in two different genes, and one had two variants (one P in *RTEL1* and one LP in *ERCC2*). The affected genes were *RNASEL* (3.5%, 12/341), *BLM* (2%; 7/341), *FAH* (2%), *PDE11A* (1.8; 6/341), *ERCC2* (1.8%), *ERCC3* (1.5%; 5/341), *MC1R* (0.9%; 3/341), *RTEL1* (1.2%, 4/341)*, POLD1* (0.6%; 2/341), *TMC6* (0.6); as well as in *CYP21A2, FANCE, MITF, ERCC6, PTCH2,* and *SBDS* genes (0.3%; 1/341). The frequent pathogenic monoallelic variant *BLM p*.Tyr736Leufs*5 found in seven participants is a well-known common founder allele in the Ashkenazi Jewish population and is associated with Bloom syndrome.

**TABLE 3:** Pathogenic and likely pathogenic alterations with unknown risk in 341 participants.

Frequency (n, %)	Gene	Type of change	Transcript	Exon	cDNA change	Protein change	Zygosity	Variant interpretation
5 (1.46)	*RNASEL*	FI	NM_021133	2	c.1441_1442insCTGCA	*p*.Tyr481Serfs*14	Heterozygous	*p*
7 (2.05)	*RNASEL*	FD	NM_021133	2	c.471_474del	*p*.Lys158Argfs*6	Heterozygous	*p*
7 (2.05)	*BLM*	FI	NM_000057	10	c.2206dupT	*p*.Tyr736Leufs*5	Heterozygous	*p*
7 (2.05)	*FAH*	Non-syn	NM_000137	9	c.782C>T	*p*.Pro261Leu	Heterozygous	*p*
6 (1.75)	*PDE11A*	FD	NM_016953	1	c.171delT	*p*.Thr58Profs*41	Heterozygous	*p*
2 (0.58)	*ERCC2*	splicing	NM_000400	7	c.594 + 2_594+5del	-	Heterozygous	*p*
4 (1.17)	*ERCC2*	Non-syn	NM_000400	22	c.2150C>G	*p*.Ala717GLy	Heterozygous	LP
5 (1.46)	*ERCC3*	stopgain	NM_000122	3	c.325C>T	*p*.Arg109*	Heterozygous	*p*
3 (0.87)	*MC1R*	stopgain	NM_002386	1	c.456C>A	*p*.Tyr152*	Heterozygous	*p*
1 (0.29)	*RTEL1*	stopgain	NM_016434	30	c.2920C>T	*p*.Arg974*	Heterozygous	*p*
2 (0.58)	*RTEL1*	Non-syn	NM_001,283,009	34	c.3791G>A	*p*.Arg1264His	Heterozygous	*p*
1 (0.29)	*RTEL1*	FD	NM_016434	32	c.3222delG	*p*.Gly1075Alafs*9	Heterozygous	*p*
2 (0.58)	*POLD1*	stopgain	NM_001,256,849	5	c.583C>T	*p*.Arg195*	Heterozygous	*p*
2 (0.58)	*TMC6*	FD	NM_007267	6	c.535_536del	*p*.Lys181GLufs*131	Heterozygous	*p*
1 (0.29)	*CYP21A2*	Non-syn	NM_000500	7	c.850 A>G	*p*.Met284Val	Heterozygous	LP
1 (0.29)	*FANCE*	Non-syn	NM_021922	5	c.1111C>T	*p*.Arg371Trp	Heterozygous	*p*
1 (0.29)	*MITF*	FI	NM_006722	1	c.4_7dup	*p*.His3Argfs*4	Heterozygous	*p*
1 (0.29)	*ERCC6*	stopgain	NM_170,753	2	c.C483G	*p*.Tyr161*	Heterozygous	*p*
1 (0.29)	*PTCH2*	FD	NM_003738	3	c.372_373del	*p*.Asn125Hisfs*19	Heterozygous	*p*
1 (0.29)	*SBDS*	splicing	NM_016038.2	2	c.258 + 2 T>C	-	Heterozygous	*p*

Non-syn, non-synonymous SNV, FI, frameshift insertion; FD, frameshift deletion, *p,* pathogenic, LP, likely pathogenic.

## Discussion

Individuals of Ashkenazi Jewish ancestry have been identified as a population with a distinctive genetic background, characterized by a higher prevalence of certain pathogenic variants associated with susceptibility to both chronic and rare diseases, as evidenced by a relatively high frequency of alleles associated with breast and ovarian cancer (Ostrer, 2001; Xiao and Lauschke, 2021). We aimed to evaluate the germline cancer susceptibility mutation landscape in the AJ diaspora in Mexico. The landscape of germline pathogenic variants present in extensive panels of cancer associated genes have not been evaluated in recent generations of women of Ashkenazi Jewish origin, in particular in those populations after migration to Latin America. Moreover, most of the genetic studies on the AJ population are conducted in patients with a confirmed diagnosis of cancer and therefore, the reported frequency of founder pathogenic germline variants could be mainly influenced by the high proportion of inherited disease cases and may be underestimated in the unaffected AJ population. Population-based screening studies in AJ women without cancer diagnosis have reported *BRCA1/2* mutation frequencies of 1.1%–2.9% (Metcalfe et al., 2010; Manchanda et al., 2015; 2020). Furthermore, the allelic composition of cancer susceptibility variants in genes distinct from *BRCA1/2* is generally not evaluated in healthy populations at higher risk. As a result, it is possible that an important proportion of AJ germline carriers is not detected, especially in low- and middle-income countries where access to genetic counseling and germline testing is still restricted.

We showed that self-identification as AJ and the ancestry genetic composition agreed sufficiently to characterize AJ population in Mexico (Mathieson and Scally, 2020). As expected, admixture of genetic ancestries was present. However, a high proportion of participants (92%) shared ≥70% of the evaluated alleles with populations located in Europe. These combined results provide evidence that endogamy in the AJ population in Mexico is still persistent as confirmed by social studies showing that bicultural marriages were scarce among Jewish immigrants, as well as among the first generations born in Mexican territory (Goldsmit Brindis, 2010).

The proportion of AJ individuals carrying *p* or LP germline variants identified (26.7%) confirms the persistent elevated risk for chronic diseases, including cancer among AJ communities in Mexico as reported in a prior publication on this population (Morgenstern-Kaplan et al., 2022). Sixteen percent of the participants carried a *p* variant in a high-risk cancer gene. All *p* variants found correspond to founder mutations previously described in the AJ population, which have not been reported in the Mexican non-Jewish population (Ellis et al., 1998; Shaag et al., 2005) and were detected in individuals without personal history of cancer. Pathogenic and LP variants were significantly associated with younger age confirming the elevated prevalence of germline genetic variants in the AJ population and the need for timely genetic testing and counseling before disease onset. Interestingly, several reports have shown that almost 50% of Jewish participants with cancer that harbor a high-risk pathogenic variant have no family history (Walsh et al., 2017; Tennen et al., 2020).

In this work, we did not detect P or LP variants in *BRCA1* or *BRCA2* genes. However, *CHEK2* LP variants were detected at a frequency of 2.9%. This frequency is not higher than those found in other previous reports for AJ breast cancer patients but slightly higher for cancer-free AJ women (Shaag et al., 2005). For cancer-free AJ women the probability of having a pathogenic mutation in *CHEK2* is 1.4%–1.7% and the chance for a mutation in *BRCA1* or *BRCA2* is less than 2.5% (Walsh et al., 2017). This result highlights the importance of comprehensive genetic testing with a multigene panel to identify *CHEK2* alterations, as well as private mutations in susceptibility genes for AJ women with no founder or *p* variant in *BRCA1* or *BRCA2*. In addition, further evidence involving treatment decisions strongly reinforces consideration for *CHEK2* testing. A recent study has shown poorer clinical outcomes for breast cancer patients diagnosed with invasive, early-onset breast cancer who carry a *CHEK2* pathogenic variant (Greville-Heygate et al., 2020). These results also echo the recommendations regarding prudent use of consumer directed genetic tests targeting high-risk populations including exclusively highly prevalent founder pathogenic variants. For example, the FDA-Authorized, Direct-To-Consumer BRCA Test commercialized by 23andMe^Ⓡ^ only identifies three *BRCA1 p* variants common in people of Ashkenazi Jewish ancestry. A negative result of this test must lead to complementary testing.

Among the recognized risk factors for breast cancer, the participants reported greater current alcohol consumption compared with the overall prevalence of alcohol consumption in Mexican women (64 percent *versus* 39 percent, respectively) (ENCODAT, 2017). In 2020, 1.3% (range 0.7%–2.1%) of all cancer cases diagnosed in women in Mexico were attributed to alcohol consumption (Rumgay et al., 2021). Considering the underlying elevated cancer risk AJ populations have, additional efforts promoting reduction of alcohol consumption should be envisioned in this community.

Interpretation of current results should consider some limitations in this study. Despite our stringent selection criteria to include a fair representation sample of the AJ community in Mexico, participants identified were born and lived in the central capital of the country and therefore do not fully represent AJ women from other regions of the country. Since in this study we investigated volunteers without medical indication, it is possible that there is a recruitment bias toward low-risk participants, as high-risk individuals may have already been analyzed by commercial laboratory testing. This may explain why common founder mutations in *BRCA1/2* were not detected in this population. Despite the vast size of our gene panel, this strategy is not able to identify variants in promoters or remote regulatory regions, or those present in sites involved in mRNA processing such as 5′- and 3′-UTRs. Intronic variants that may affect splicing are also not covered. It is possible that these non-coding variants may contribute to the pathogenic allele pool of this population as has been shown in other reports (Hansmann et al., 2012; Burke et al., 2018; Evans et al., 2018; Montalban et al., 2019). Additional investigations involving whole genome sequencing and functional analysis are required to better understand the impact and composition of pathogenic genetic variation in this population.

This is the first study that estimates the prevalence of P and LP alleles in Mexican women who self-identify as AJ, showing an elevated frequency (26.7%) of participants with P or LP germline variants in cancer susceptibility genes, of which 14% were in high-risk genes. In the study sample of Ashkenazi Jewish individuals, we did not identify common pathogenic mutations in the *BRCA1/2* genes but found high-penetrance mutations in other cancer associated genes such as *MSH6, APC* and *MSH2.* Pathogenic variants in HBOC moderate-risk genes such as *CHEK2* were frequent in study participants without personal history of cancer. These findings need to be further explored as to better describe the cancer genetic risk of women who identify as AJ in Mexico and develop targeted cancer prevention programs focused both on genetic screening and modifiable risk factors such as reduction of alcohol consumption.

## Data Availability

The sequencing files can be accessed in the Sequence Reference Archive with the accession ID PRJNA914196. https://www.ncbi.nlm.nih.gov/bioproject/PRJNA914196/.

## Supplementary material

SUPPLEMENTARY FIGURE S1 Principal component analysis showing ancestral composition of the reference populations from 1000 G using 57 pruned variants selected from a panel of 104 ancestry informative SNPs genotyped using the MassARRAY System (Agena Bioscience). POP, Populations; GBR, British; FIN, Finnish; IBS Iberian; CEU, Northern and Western European; TSI, Toscani; MXL, Mexican; PEL, Peruvian; CLM, Colombian; PUR, Puerto Rican; YRI, Yoruba.

SUPPLEMENTARY TABLE S1 Ancestry components of the participants.

SUPPLEMENTARY TABLE S2 Demographic variables for the 431 AJ participants according to presence of at least one pathogenic or likely pathogenic germline variant. For the Tobacco /Alcohol consumption variable, current smokers and current drinkers included those women who reported daily smoking or drinking at time of recruitment. Pregnancy variable included women who had at least one full term pregnancy. Contraceptive use refers to consecutively use during the last year.

SUPPLEMENTARY TABLE S3 Variants with unknown clinical significance in genes associated with hereditary breast and ovarian cancer syndrome or other cancers.
