# Supplementary material for: Evaluation of genetic alterations in hereditary cancer susceptibility genes in the Ashkenazi Jewish women community of Mexico
Source: Front Genet. 2023 Feb 10;14:1094260. doi: 10.3389/fgene.2023.1094260 (PMC9950094; doi:10.3389/fgene.2023.1094260)
Supplement: Supplementary file 1 [file Table1.docx]

| ID | latino_admixture | EUR_admixture | AFR_admixture | Groups |
| --- | --- | --- | --- | --- |
| JA01 | 0.063276 | 0.936714 | 1.00E-05 | PJA:EUR>70% |
| JA02 | 0.137324 | 0.772783 | 0.089892 | PJA:EUR>70% |
| JA03 | 0.098277 | 0.852049 | 0.049674 | PJA:EUR>70% |
| JA04 | 0.074274 | 0.74761 | 0.178116 | PJA:EUR>70% |
| JA05 | 0.055224 | 0.895378 | 0.049398 | PJA:EUR>70% |
| JA06 | 0.053448 | 0.871123 | 0.075429 | PJA:EUR>70% |
| JA07 | 0.129269 | 0.769563 | 0.101168 | PJA:EUR>70% |
| JA08 | 0.147546 | 0.76298 | 0.089473 | PJA:EUR>70% |
| JA09 | 0.162903 | 0.736435 | 0.100661 | PJA:EUR>70% |
| JA10 | 0.123697 | 0.778594 | 0.097709 | PJA:EUR>70% |
| JA11 | 0.112799 | 0.887191 | 1.00E-05 | PJA:EUR>70% |
| JA12 | 0.02997 | 0.846357 | 0.123673 | PJA:EUR>70% |
| JA13 | 0.16658 | 0.808127 | 0.025293 | PJA:EUR>70% |
| JA14 | 0.124663 | 0.838827 | 0.03651 | PJA:EUR>70% |
| JA15 | 0.013731 | 0.812858 | 0.173412 | PJA:EUR>70% |
| JA16 | 0.08799 | 0.869463 | 0.042548 | PJA:EUR>70% |
| JA17 | 0.045235 | 0.844406 | 0.110359 | PJA:EUR>70% |
| JA18 | 0.097289 | 0.800063 | 0.102648 | PJA:EUR>70% |
| JA19 | 1.00E-05 | 0.836682 | 0.163308 | PJA:EUR>70% |
| JA20 | 0.040342 | 0.864979 | 0.09468 | PJA:EUR>70% |
| JA21 | 0.139418 | 0.739993 | 0.120588 | PJA:EUR>70% |
| JA22 | 0.08885 | 0.708733 | 0.202417 | PJA:EUR>70% |
| JA23 | 0.118628 | 0.847081 | 0.034291 | PJA:EUR>70% |
| JA24 | 1.00E-05 | 0.939468 | 0.060522 | PJA:EUR>70% |
| JA25 | 0.056806 | 0.943184 | 1.00E-05 | PJA:EUR>70% |
| JA26 | 0.092389 | 0.877411 | 0.0302 | PJA:EUR>70% |
| JA27 | 0.008201 | 0.991364 | 0.000435 | PJA:EUR>70% |
| JA28 | 0.047431 | 0.940259 | 0.01231 | PJA:EUR>70% |
| JA29 | 0.112809 | 0.817871 | 0.069319 | PJA:EUR>70% |
| JA30 | 0.10735 | 0.77062 | 0.12203 | PJA:EUR>70% |
| JA31 | 0.179044 | 0.820946 | 1.00E-05 | PJA:EUR>70% |
| JA32 | 1.00E-05 | 0.927951 | 0.072039 | PJA:EUR>70% |
| JA33 | 0.165147 | 0.724908 | 0.109946 | PJA:EUR>70% |
| JA34 | 0.087927 | 0.685095 | 0.226978 | PJA:EUR<70% |
| JA35 | 0.084492 | 0.915498 | 1.00E-05 | PJA:EUR>70% |
| JA36 | 0.06407 | 0.901856 | 0.034075 | PJA:EUR>70% |
| JA37 | 0.137352 | 0.862638 | 1.00E-05 | PJA:EUR>70% |
| JA38 | 0.114321 | 0.834044 | 0.051635 | PJA:EUR>70% |
| JA39 | 0.046299 | 0.819694 | 0.134007 | PJA:EUR>70% |
| JA40 | 0.167996 | 0.658796 | 0.173208 | PJA:EUR<70% |
| JA41 | 0.125082 | 0.874908 | 1.00E-05 | PJA:EUR>70% |
| JA42 | 0.077944 | 0.802556 | 0.1195 | PJA:EUR>70% |
| JA43 | 0.007072 | 0.934239 | 0.058689 | PJA:EUR>70% |
| JA44 | 1.00E-05 | 0.994365 | 0.005625 | PJA:EUR>70% |
| JA45 | 0.247966 | 0.74916 | 0.002874 | PJA:EUR>70% |
| JA46 | 0.133522 | 0.650143 | 0.216335 | PJA:EUR<70% |
| JA47 | 0.254717 | 0.632413 | 0.11287 | PJA:EUR<70% |
| JA48 | 0.061431 | 0.844593 | 0.093976 | PJA:EUR>70% |
| JA49 | 1.00E-05 | 0.842642 | 0.157348 | PJA:EUR>70% |
| JA50 | 0.020329 | 0.875293 | 0.104378 | PJA:EUR>70% |
| JA51 | 0.093882 | 0.898952 | 0.007166 | PJA:EUR>70% |
| JA52 | 0.01342 | 0.943785 | 0.042795 | PJA:EUR>70% |
| JA53 | 1.00E-05 | 0.899224 | 0.100766 | PJA:EUR>70% |
| JA54 | 0.053655 | 0.916183 | 0.030162 | PJA:EUR>70% |
| JA55 | 0.001335 | 0.893491 | 0.105173 | PJA:EUR>70% |
| JA56 | 0.022373 | 0.897546 | 0.08008 | PJA:EUR>70% |
| JA57 | 0.160244 | 0.733475 | 0.106281 | PJA:EUR>70% |
| JA58 | 0.35422 | 0.526671 | 0.119109 | PJA:EUR<70% |
| JA59 | 0.05266 | 0.825978 | 0.121361 | PJA:EUR>70% |
| JA60 | 0.177878 | 0.725129 | 0.096993 | PJA:EUR>70% |
| JA61 | 0.154522 | 0.811076 | 0.034402 | PJA:EUR>70% |
| JA62 | 0.016324 | 0.966183 | 0.017493 | PJA:EUR>70% |
| JA63 | 0.082929 | 0.84117 | 0.075901 | PJA:EUR>70% |
| JA64 | 1.00E-05 | 0.770093 | 0.229897 | PJA:EUR>70% |
| JA65 | 0.037585 | 0.810799 | 0.151616 | PJA:EUR>70% |
| JA66 | 1.00E-05 | 0.888491 | 0.111499 | PJA:EUR>70% |
| JA67 | 0.070628 | 0.86538 | 0.063992 | PJA:EUR>70% |
| JA68 | 0.065093 | 0.925224 | 0.009683 | PJA:EUR>70% |
| JA69 | 1.00E-05 | 0.99998 | 1.00E-05 | PJA:EUR>70% |
| JA70 | 0.084303 | 0.638172 | 0.277526 | PJA:EUR<70% |
| PJA_71 | 0.203759 | 0.66523 | 0.131011 | PJA:EUR<70% |
| PJA_72 | 0.100016 | 0.813757 | 0.086228 | PJA:EUR>70% |
| PJA_73 | 1.00E-05 | 0.861555 | 0.138435 | PJA:EUR>70% |
| PJA_74 | 0.222678 | 0.777312 | 1.00E-05 | PJA:EUR>70% |
| PJA_75 | 0.159023 | 0.686769 | 0.154208 | PJA:EUR<70% |
| PJA_76 | 0.036351 | 0.91806 | 0.045589 | PJA:EUR>70% |
| PJA_77 | 0.137645 | 0.862345 | 1.00E-05 | PJA:EUR>70% |
| PJA_78 | 0.130642 | 0.869348 | 1.00E-05 | PJA:EUR>70% |
| PJA_79 | 1.00E-05 | 0.893182 | 0.106808 | PJA:EUR>70% |
| PJA_80 | 0.0137 | 0.940071 | 0.046229 | PJA:EUR>70% |
| PJA_81 | 0.048334 | 0.874703 | 0.076963 | PJA:EUR>70% |
| PJA_82 | 1.00E-05 | 0.844489 | 0.155501 | PJA:EUR>70% |
| PJA_83 | 0.095576 | 0.820635 | 0.083789 | PJA:EUR>70% |
| PJA_84 | 0.046748 | 0.912954 | 0.040299 | PJA:EUR>70% |
| PJA_85 | 0.043937 | 0.906698 | 0.049365 | PJA:EUR>70% |
| PJA_86 | 1.00E-05 | 0.751901 | 0.248089 | PJA:EUR>70% |
| PJA_87 | 0.079655 | 0.744386 | 0.175959 | PJA:EUR>70% |
| PJA_88 | 0.045947 | 0.81421 | 0.139844 | PJA:EUR>70% |
| PJA_89 | 0.150925 | 0.849065 | 1.00E-05 | PJA:EUR>70% |
| PJA_90 | 1.00E-05 | 0.78706 | 0.21293 | PJA:EUR>70% |
| PJA_91 | 0.142964 | 0.73054 | 0.126496 | PJA:EUR>70% |
| PJA_92 | 0.08616 | 0.816605 | 0.097236 | PJA:EUR>70% |
| PJA_93 | 0.091412 | 0.854142 | 0.054446 | PJA:EUR>70% |
| PJA_94 | 0.06542 | 0.812993 | 0.121587 | PJA:EUR>70% |
| PJA_95 | 0.020541 | 0.893686 | 0.085773 | PJA:EUR>70% |
| PJA_96 | 0.040677 | 0.885748 | 0.073575 | PJA:EUR>70% |
| PJA_97 | 1.00E-05 | 0.947555 | 0.052435 | PJA:EUR>70% |
| PJA_98 | 0.1407 | 0.795579 | 0.063721 | PJA:EUR>70% |
| PJA_99 | 1.00E-05 | 0.940177 | 0.059813 | PJA:EUR>70% |
| PJA_100 | 0.026788 | 0.881746 | 0.091466 | PJA:EUR>70% |
| PJA_101 | 0.018286 | 0.829278 | 0.152437 | PJA:EUR>70% |
| PJA_102 | 0.073248 | 0.787357 | 0.139395 | PJA:EUR>70% |
| PJA_103 | 0.243418 | 0.636342 | 0.12024 | PJA:EUR<70% |
| PJA_104 | 0.063338 | 0.894003 | 0.042659 | PJA:EUR>70% |
| PJA_105 | 1.00E-05 | 0.885306 | 0.114684 | PJA:EUR>70% |
| PJA_106 | 0.157216 | 0.830011 | 0.012773 | PJA:EUR>70% |
| PJA_107 | 0.007585 | 0.8822 | 0.110216 | PJA:EUR>70% |
| PJA_108 | 0.112997 | 0.719768 | 0.167235 | PJA:EUR>70% |
| PJA_109 | 0.006325 | 0.820112 | 0.173563 | PJA:EUR>70% |
| PJA_110 | 0.097325 | 0.806718 | 0.095957 | PJA:EUR>70% |
| PJA_111 | 1.00E-05 | 0.91542 | 0.08457 | PJA:EUR>70% |
| PJA_112 | 0.126368 | 0.783081 | 0.090551 | PJA:EUR>70% |
| PJA_113 | 0.032673 | 0.907516 | 0.059811 | PJA:EUR>70% |
| PJA_114 | 0.218845 | 0.722719 | 0.058436 | PJA:EUR>70% |
| PJA_115 | 1.00E-05 | 0.84413 | 0.15586 | PJA:EUR>70% |
| PJA_116 | 0.076816 | 0.761876 | 0.161308 | PJA:EUR>70% |
| PJA_117 | 0.209233 | 0.678753 | 0.112014 | PJA:EUR<70% |
| PJA_118 | 0.062516 | 0.808471 | 0.129013 | PJA:EUR>70% |
| PJA_119 | 0.094856 | 0.782916 | 0.122229 | PJA:EUR>70% |
| PJA_120 | 0.080407 | 0.815877 | 0.103716 | PJA:EUR>70% |
| PJA_121 | 0.066101 | 0.881287 | 0.052611 | PJA:EUR>70% |
| PJA_122 | 0.122596 | 0.877394 | 1.00E-05 | PJA:EUR>70% |
| PJA_123 | 0.089879 | 0.910111 | 1.00E-05 | PJA:EUR>70% |
| PJA_124 | 0.084978 | 0.883678 | 0.031344 | PJA:EUR>70% |
| PJA_125 | 0.088581 | 0.818157 | 0.093262 | PJA:EUR>70% |
| PJA_126 | 1.00E-05 | 0.803423 | 0.196567 | PJA:EUR>70% |
| PJA_127 | 0.091334 | 0.90855 | 0.000116 | PJA:EUR>70% |
| PJA_128 | 0.059258 | 0.844883 | 0.095859 | PJA:EUR>70% |
| PJA_129 | 0.115155 | 0.870743 | 0.014102 | PJA:EUR>70% |
| PJA_130 | 0.10364 | 0.847269 | 0.049091 | PJA:EUR>70% |
| PJA_131 | 0.180515 | 0.748767 | 0.070719 | PJA:EUR>70% |
| PJA_132 | 0.170185 | 0.791014 | 0.038802 | PJA:EUR>70% |
| PJA_133 | 0.028413 | 0.803252 | 0.168335 | PJA:EUR>70% |
| PJA_134 | 0.023648 | 0.823126 | 0.153225 | PJA:EUR>70% |
| PJA_135 | 0.058035 | 0.749344 | 0.192621 | PJA:EUR>70% |
| PJA_136 | 0.09916 | 0.716758 | 0.184082 | PJA:EUR>70% |
| PJA_137 | 0.079946 | 0.828047 | 0.092008 | PJA:EUR>70% |
| PJA_138 | 0.11832 | 0.793394 | 0.088286 | PJA:EUR>70% |
| PJA_139 | 0.114659 | 0.862373 | 0.022967 | PJA:EUR>70% |
| PJA_140 | 0.109166 | 0.744305 | 0.146529 | PJA:EUR>70% |
| PJA_141 | 0.087808 | 0.828547 | 0.083645 | PJA:EUR>70% |
| PJA_142 | 0.212723 | 0.689501 | 0.097776 | PJA:EUR<70% |
| PJA_143 | 0.158002 | 0.725704 | 0.116294 | PJA:EUR>70% |
| PJA_144 | 0.093881 | 0.850076 | 0.056043 | PJA:EUR>70% |
| PJA_145 | 0.151217 | 0.737061 | 0.111722 | PJA:EUR>70% |
| PJA_146 | 0.162378 | 0.837612 | 1.00E-05 | PJA:EUR>70% |
| PJA_147 | 0.054711 | 0.876377 | 0.068912 | PJA:EUR>70% |
| PJA_149 | 1.00E-05 | 0.776461 | 0.223529 | PJA:EUR>70% |
| PJA_151 | 1.00E-05 | 0.895069 | 0.104921 | PJA:EUR>70% |
| PJA_152 | 1.00E-05 | 0.925698 | 0.074292 | PJA:EUR>70% |
| PJA_153 | 0.096112 | 0.801221 | 0.102667 | PJA:EUR>70% |
| PJA_154 | 0.131357 | 0.832205 | 0.036437 | PJA:EUR>70% |
| PJA_155 | 0.119801 | 0.769518 | 0.110681 | PJA:EUR>70% |
| PJA_156 | 0.124554 | 0.855273 | 0.020173 | PJA:EUR>70% |
| PJA_158 | 0.027914 | 0.774203 | 0.197883 | PJA:EUR>70% |
| PJA_159 | 1.00E-05 | 0.877794 | 0.122196 | PJA:EUR>70% |
| PJA_160 | 0.057883 | 0.9064 | 0.035717 | PJA:EUR>70% |
| PJA_161 | 0.048686 | 0.853245 | 0.098069 | PJA:EUR>70% |
| PJA_162 | 0.107135 | 0.747909 | 0.144956 | PJA:EUR>70% |
| PJA_163 | 0.157814 | 0.842176 | 1.00E-05 | PJA:EUR>70% |
| PJA_164 | 0.159647 | 0.840343 | 1.00E-05 | PJA:EUR>70% |
| PJA_165 | 1.00E-05 | 0.895969 | 0.104021 | PJA:EUR>70% |
| PJA_166 | 0.13498 | 0.816023 | 0.048997 | PJA:EUR>70% |
| PJA_167 | 0.107284 | 0.855488 | 0.037227 | PJA:EUR>70% |
| PJA_168 | 0.102581 | 0.897409 | 1.00E-05 | PJA:EUR>70% |
| PJA_169 | 0.066532 | 0.784993 | 0.148475 | PJA:EUR>70% |
| PJA_170 | 0.12216 | 0.865257 | 0.012583 | PJA:EUR>70% |
| PJA_171 | 0.101589 | 0.898401 | 1.00E-05 | PJA:EUR>70% |
| PJA_172 | 0.146835 | 0.792357 | 0.060808 | PJA:EUR>70% |
| PJA_173 | 1.00E-05 | 0.886599 | 0.113391 | PJA:EUR>70% |
| PJA_174 | 1.00E-05 | 0.839759 | 0.160231 | PJA:EUR>70% |
| PJA_176 | 0.038456 | 0.961534 | 1.00E-05 | PJA:EUR>70% |
| PJA_177 | 0.272132 | 0.727858 | 1.00E-05 | PJA:EUR>70% |
| PJA_178 | 0.012009 | 0.845004 | 0.142987 | PJA:EUR>70% |
| PJA_179 | 0.079591 | 0.789469 | 0.13094 | PJA:EUR>70% |
| PJA_180 | 0.147253 | 0.574171 | 0.278576 | PJA:EUR<70% |
| PJA_181 | 0.026855 | 0.914398 | 0.058747 | PJA:EUR>70% |
| PJA_182 | 0.097648 | 0.864206 | 0.038146 | PJA:EUR>70% |
| PJA_183 | 0.157426 | 0.830258 | 0.012315 | PJA:EUR>70% |
| PJA_184 | 1.00E-05 | 0.928837 | 0.071153 | PJA:EUR>70% |
| PJA_185 | 0.115523 | 0.739712 | 0.144765 | PJA:EUR>70% |
| PJA_186 | 0.1283 | 0.792337 | 0.079362 | PJA:EUR>70% |
| PJA_187 | 0.110134 | 0.884265 | 0.005601 | PJA:EUR>70% |
| PJA_188 | 0.048081 | 0.857124 | 0.094795 | PJA:EUR>70% |
| PJA_189 | 0.21204 | 0.78795 | 1.00E-05 | PJA:EUR>70% |
| PJA_190 | 1.00E-05 | 0.836288 | 0.163702 | PJA:EUR>70% |
| PJA_191 | 0.057783 | 0.783923 | 0.158294 | PJA:EUR>70% |
| PJA_192 | 0.151434 | 0.775697 | 0.072869 | PJA:EUR>70% |
| PJA_193 | 0.258035 | 0.622471 | 0.119494 | PJA:EUR<70% |
| PJA_194 | 0.156398 | 0.753943 | 0.089659 | PJA:EUR>70% |
| PJA_195 | 0.087468 | 0.912522 | 1.00E-05 | PJA:EUR>70% |
| PJA_196 | 0.108304 | 0.826105 | 0.06559 | PJA:EUR>70% |
| PJA_197 | 1.00E-05 | 0.860351 | 0.139639 | PJA:EUR>70% |
| PJA_199 | 0.257919 | 0.629963 | 0.112119 | PJA:EUR<70% |
| PJA_201 | 0.057529 | 0.891795 | 0.050676 | PJA:EUR>70% |
| PJA_202 | 0.065411 | 0.8118 | 0.122788 | PJA:EUR>70% |
| PJA_203 | 0.08347 | 0.865447 | 0.051083 | PJA:EUR>70% |
| PJA_204 | 0.076703 | 0.879982 | 0.043315 | PJA:EUR>70% |
| PJA_205 | 0.065435 | 0.897896 | 0.036669 | PJA:EUR>70% |
| PJA_206 | 0.124031 | 0.781219 | 0.09475 | PJA:EUR>70% |
| PJA_207 | 0.078506 | 0.921484 | 1.00E-05 | PJA:EUR>70% |
| PJA_208 | 0.208741 | 0.791249 | 1.00E-05 | PJA:EUR>70% |
| PJA_209 | 0.152245 | 0.843457 | 0.004298 | PJA:EUR>70% |
| PJA_210 | 0.09516 | 0.721335 | 0.183505 | PJA:EUR>70% |
| PJA_211 | 0.062643 | 0.809627 | 0.12773 | PJA:EUR>70% |
| PJA_212 | 0.01236 | 0.98763 | 1.00E-05 | PJA:EUR>70% |
| PJA_213 | 0.139099 | 0.860891 | 1.00E-05 | PJA:EUR>70% |
| PJA_214 | 0.076753 | 0.766084 | 0.157163 | PJA:EUR>70% |
| PJA_215 | 0.10106 | 0.785859 | 0.113081 | PJA:EUR>70% |
| PJA_216 | 0.132739 | 0.785475 | 0.081785 | PJA:EUR>70% |
| PJA_217 | 1.00E-05 | 0.917151 | 0.082839 | PJA:EUR>70% |
| PJA_218 | 0.176611 | 0.817927 | 0.005463 | PJA:EUR>70% |
| PJA_219 | 0.064723 | 0.79305 | 0.142226 | PJA:EUR>70% |
| PJA_220 | 0.211899 | 0.788091 | 1.00E-05 | PJA:EUR>70% |
| PJA_221 | 0.14864 | 0.785748 | 0.065613 | PJA:EUR>70% |
| PJA_222 | 0.106054 | 0.877379 | 0.016566 | PJA:EUR>70% |
| PJA_223 | 0.085399 | 0.858644 | 0.055958 | PJA:EUR>70% |
| PJA_224 | 0.072234 | 0.927756 | 1.00E-05 | PJA:EUR>70% |
| PJA_225 | 0.048381 | 0.848547 | 0.103072 | PJA:EUR>70% |
| PJA_226 | 0.070287 | 0.868947 | 0.060767 | PJA:EUR>70% |
| PJA_227 | 0.080115 | 0.831034 | 0.088851 | PJA:EUR>70% |
| PJA_228 | 1.00E-05 | 0.923926 | 0.076064 | PJA:EUR>70% |
| PJA_229 | 0.033844 | 0.826982 | 0.139174 | PJA:EUR>70% |
| PJA_230 | 0.100459 | 0.886462 | 0.013078 | PJA:EUR>70% |
| PJA_231 | 0.151652 | 0.848338 | 1.00E-05 | PJA:EUR>70% |
| PJA_232 | 0.045261 | 0.832875 | 0.121864 | PJA:EUR>70% |
| PJA_233 | 0.093457 | 0.748225 | 0.158318 | PJA:EUR>70% |
| PJA_234 | 0.125901 | 0.722723 | 0.151376 | PJA:EUR>70% |
| PJA_235 | 0.070667 | 0.886938 | 0.042395 | PJA:EUR>70% |
| PJA_236 | 1.00E-05 | 0.949901 | 0.050089 | PJA:EUR>70% |
| PJA_237 | 0.042605 | 0.88119 | 0.076205 | PJA:EUR>70% |
| PJA_238 | 0.091336 | 0.772869 | 0.135795 | PJA:EUR>70% |
| PJA_239 | 1.00E-05 | 0.932944 | 0.067046 | PJA:EUR>70% |
| PJA_240 | 0.147508 | 0.695953 | 0.156539 | PJA:EUR<70% |
| PJA_242 | 0.108513 | 0.808151 | 0.083336 | PJA:EUR>70% |
| PJA_243 | 0.23707 | 0.598294 | 0.164636 | PJA:EUR<70% |
| PJA_244 | 0.007756 | 0.96487 | 0.027373 | PJA:EUR>70% |
| PJA_245 | 0.050912 | 0.893362 | 0.055726 | PJA:EUR>70% |
| PJA_246 | 0.007382 | 0.904818 | 0.087799 | PJA:EUR>70% |
| PJA_247 | 0.026364 | 0.912044 | 0.061592 | PJA:EUR>70% |
| PJA_248 | 0.156572 | 0.825477 | 0.017951 | PJA:EUR>70% |
| PJA_249 | 0.029932 | 0.801089 | 0.168979 | PJA:EUR>70% |
| PJA_250 | 0.072471 | 0.858781 | 0.068748 | PJA:EUR>70% |
| PJA_251 | 0.102382 | 0.880734 | 0.016884 | PJA:EUR>70% |
| PJA_252 | 0.047484 | 0.94277 | 0.009746 | PJA:EUR>70% |
| PJA_253 | 1.00E-05 | 0.845029 | 0.154961 | PJA:EUR>70% |
| PJA_254 | 0.17324 | 0.82675 | 1.00E-05 | PJA:EUR>70% |
| PJA_255 | 0.045728 | 0.886285 | 0.067987 | PJA:EUR>70% |
| PJA_256 | 0.218926 | 0.660038 | 0.121036 | PJA:EUR<70% |
| PJA_257 | 0.177533 | 0.662129 | 0.160338 | PJA:EUR<70% |
| PJA_258 | 0.133956 | 0.692581 | 0.173464 | PJA:EUR<70% |
| PJA_259 | 0.228814 | 0.771176 | 1.00E-05 | PJA:EUR>70% |
| PJA_260 | 0.084197 | 0.888532 | 0.027271 | PJA:EUR>70% |
| PJA_261 | 0.126608 | 0.831127 | 0.042265 | PJA:EUR>70% |
| PJA_262 | 0.048193 | 0.913748 | 0.038059 | PJA:EUR>70% |
| PJA_263 | 0.108828 | 0.81927 | 0.071902 | PJA:EUR>70% |
| PJA_264 | 1.00E-05 | 0.87942 | 0.12057 | PJA:EUR>70% |
| PJA_265 | 0.067419 | 0.904482 | 0.028099 | PJA:EUR>70% |
| PJA_266 | 0.135081 | 0.846616 | 0.018303 | PJA:EUR>70% |
| PJA_267 | 0.116834 | 0.580627 | 0.302539 | PJA:EUR<70% |
| PJA_268 | 0.113594 | 0.742881 | 0.143526 | PJA:EUR>70% |
| PJA_269 | 0.079511 | 0.778674 | 0.141816 | PJA:EUR>70% |
| PJA_270 | 0.041511 | 0.818027 | 0.140463 | PJA:EUR>70% |
| PJA_271 | 0.19691 | 0.720518 | 0.082572 | PJA:EUR>70% |
| PJA_273 | 0.137789 | 0.84006 | 0.022151 | PJA:EUR>70% |
| PJA_274 | 0.105217 | 0.838531 | 0.056252 | PJA:EUR>70% |
| PJA_275 | 0.042479 | 0.957511 | 1.00E-05 | PJA:EUR>70% |
| PJA_276 | 1.00E-05 | 0.834418 | 0.165572 | PJA:EUR>70% |
| PJA_277 | 1.00E-05 | 0.875519 | 0.124471 | PJA:EUR>70% |
| PJA_278 | 1.00E-05 | 0.99998 | 1.00E-05 | PJA:EUR>70% |
| PJA_279 | 0.152914 | 0.847076 | 1.00E-05 | PJA:EUR>70% |
| PJA_280 | 0.017081 | 0.787645 | 0.195274 | PJA:EUR>70% |
| PJA_281 | 0.032492 | 0.967498 | 1.00E-05 | PJA:EUR>70% |
| PJA_283 | 0.080359 | 0.794081 | 0.12556 | PJA:EUR>70% |
| PJA_284 | 0.011764 | 0.877627 | 0.11061 | PJA:EUR>70% |
| PJA_285 | 1.00E-05 | 0.85526 | 0.14473 | PJA:EUR>70% |
| PJA_286 | 0.029815 | 0.81114 | 0.159045 | PJA:EUR>70% |
| PJA_287 | 0.153472 | 0.813875 | 0.032653 | PJA:EUR>70% |
| PJA_288 | 0.058326 | 0.941664 | 1.00E-05 | PJA:EUR>70% |
| PJA_289 | 0.065709 | 0.925852 | 0.00844 | PJA:EUR>70% |
| PJA_290 | 1.00E-05 | 0.857374 | 0.142616 | PJA:EUR>70% |
| PJA_291 | 0.189723 | 0.810267 | 1.00E-05 | PJA:EUR>70% |
| PJA_292 | 0.067797 | 0.879324 | 0.052879 | PJA:EUR>70% |
| PJA_294 | 0.088196 | 0.911794 | 1.00E-05 | PJA:EUR>70% |
| PJA_295 | 0.113371 | 0.824259 | 0.06237 | PJA:EUR>70% |
| PJA_296 | 0.018001 | 0.734392 | 0.247607 | PJA:EUR>70% |
| PJA_297 | 0.011061 | 0.970585 | 0.018354 | PJA:EUR>70% |
| PJA_298 | 0.009578 | 0.793667 | 0.196755 | PJA:EUR>70% |
| PJA_299 | 0.03939 | 0.824256 | 0.136353 | PJA:EUR>70% |
| PJA_300 | 0.050256 | 0.88218 | 0.067563 | PJA:EUR>70% |
| PJA_301 | 0.010497 | 0.866779 | 0.122724 | PJA:EUR>70% |
| PJA_302 | 1.00E-05 | 0.926628 | 0.073362 | PJA:EUR>70% |
| PJA_303 | 0.046373 | 0.953617 | 1.00E-05 | PJA:EUR>70% |
| PJA_304 | 0.111008 | 0.88176 | 0.007232 | PJA:EUR>70% |
| PJA_305 | 0.000121 | 0.906329 | 0.09355 | PJA:EUR>70% |
| PJA_306 | 1.00E-05 | 0.873429 | 0.126561 | PJA:EUR>70% |
| PJA_307 | 0.168134 | 0.831856 | 1.00E-05 | PJA:EUR>70% |
| PJA_309 | 0.13838 | 0.72596 | 0.13566 | PJA:EUR>70% |
| PJA_310 | 0.066062 | 0.770172 | 0.163766 | PJA:EUR>70% |
| PJA_311 | 0.067038 | 0.755679 | 0.177283 | PJA:EUR>70% |
| PJA_312 | 0.013345 | 0.773856 | 0.212799 | PJA:EUR>70% |
| PJA_313 | 0.018599 | 0.819984 | 0.161417 | PJA:EUR>70% |
| PJA_314 | 0.155674 | 0.844316 | 1.00E-05 | PJA:EUR>70% |
| PJA_315 | 0.033513 | 0.843143 | 0.123344 | PJA:EUR>70% |
| PJA_317 | 1.00E-05 | 0.99998 | 1.00E-05 | PJA:EUR>70% |
| PJA_318 | 0.04336 | 0.847609 | 0.109031 | PJA:EUR>70% |
| PJA_319 | 0.13056 | 0.845267 | 0.024172 | PJA:EUR>70% |
| PJA_320 | 0.067415 | 0.932575 | 1.00E-05 | PJA:EUR>70% |
| PJA_321 | 0.028282 | 0.809567 | 0.162151 | PJA:EUR>70% |
| PJA_322 | 0.075594 | 0.914752 | 0.009654 | PJA:EUR>70% |
| PJA_323 | 0.027499 | 0.914782 | 0.057719 | PJA:EUR>70% |
| PJA_325 | 0.013385 | 0.928989 | 0.057626 | PJA:EUR>70% |
| PJA_326 | 0.092746 | 0.734621 | 0.172632 | PJA:EUR>70% |
| PJA_327 | 0.121091 | 0.721061 | 0.157849 | PJA:EUR>70% |
| PJA_328 | 0.185348 | 0.661057 | 0.153594 | PJA:EUR<70% |
| PJA_329 | 0.045846 | 0.864417 | 0.089737 | PJA:EUR>70% |
| PJA_330 | 0.126386 | 0.816358 | 0.057256 | PJA:EUR>70% |
| PJA_331 | 0.016898 | 0.852788 | 0.130314 | PJA:EUR>70% |
| PJA_332 | 0.105626 | 0.891413 | 0.002961 | PJA:EUR>70% |
| PJA_333 | 0.082314 | 0.908571 | 0.009115 | PJA:EUR>70% |
| PJA_335 | 0.364412 | 0.588256 | 0.047332 | PJA:EUR<70% |
| PJA_336 | 1.00E-05 | 0.779371 | 0.220619 | PJA:EUR>70% |
| PJA_337 | 0.029832 | 0.875807 | 0.09436 | PJA:EUR>70% |
| PJA_338 | 0.142646 | 0.841229 | 0.016126 | PJA:EUR>70% |
| PJA_339 | 0.367688 | 0.558988 | 0.073324 | PJA:EUR<70% |
| PJA_340 | 0.223158 | 0.693725 | 0.083117 | PJA:EUR<70% |
| PJA_341 | 0.308541 | 0.691449 | 1.00E-05 | PJA:EUR<70% |
