# Supplementary material for: Evaluation of genetic alterations in hereditary cancer susceptibility genes in the Ashkenazi Jewish women community of Mexico
Source: Front Genet. 2023 Feb 10;14:1094260. doi: 10.3389/fgene.2023.1094260 (PMC9950094; doi:10.3389/fgene.2023.1094260)
Supplement: Supplementary file 2 [file Table2.docx]

| Epidemiological description | | | | |
| --- | --- | --- | --- | --- |
| Characteristic | Overall, N = 341*^1^* | Wild allele, N = 250*^1^* | Pathogenic or likely pathogenic variant, N = 91*^1^* | p-value*^2^* |
| Age | 47 (14) | 48 (15) | 45 (14) | 0.043 |
| Current BMI (Kg/m2) | 23.0 (21.0, 26.3) | 23.0 (20.9, 26.5) | 22.9 (21.2, 25.6) | 0.42 |
| 20y BMI (Kg/m2) | 20.8 (19.1, 22.8) | 20.7 (19.1, 22.9) | 21.3 (19.7, 22.5) | 0.13 |
| Educational level |  |  |  | 0.10 |
| Illiterate | 0 (0%) | 0 (0%) | 0 (0%) |  |
| Primary | 0 (0%) | 0 (0%) | 0 (0%) |  |
| Secondary | 3 (0.9%) | 1 (0.4%) | 2 (2.2%) |  |
| Superior/Technical | 28 (8.2%) | 23 (9.2%) | 5 (5.6%) |  |
| University | 179 (53%) | 137 (55%) | 42 (47%) |  |
| Post university | 130 (38%) | 89 (36%) | 41 (46%) |  |
| No data available | 1 | 0 | 1 |  |
| Personal history cancer |  |  |  | 0.25 |
| yes | 50 (15%) | 41 (16%) | 9 (9.9%) |  |
| No | 289 (85%) | 207 (83%) | 82 (90%) |  |
| Not known | 2 (0.6%) | 2 (0.8%) | 0 (0%) |  |
| Pregnancy | 289 (85%) | 211 (84%) | 78 (86%) | 0.77 |
| Age first child | 26 (24, 28) | 25 (23, 28) | 26 (25, 27) | 0.42 |
| No data available | 55 | 42 | 13 |  |
| Contraceptive use | 301 (88%) | 221 (88%) | 80 (88%) | 0.90 |
| Tobacco consumption |  |  |  | 0.32 |
| Never | 220 (65%) | 167 (67%) | 53 (58%) |  |
| Former | 79 (23%) | 55 (22%) | 24 (26%) |  |
| Current | 42 (12%) | 28 (11%) | 14 (15%) |  |
| Alcohol consumption |  |  |  | 0.80 |
| Current | 218 (64%) | 159 (64%) | 59 (65%) |  |
| Former | 6 (1.8%) | 4 (1.6%) | 2 (2.2%) |  |
| Never | 117 (34%) | 87 (35%) | 30 (33%) |  |
| *^1^* Mean (SD); Median (IQR); n (%)  *^2^* Welch Two Sample t-test; Fisher's exact test; Pearson's Chi-squared test | | | | |
