# Supplementary material for: Evaluation of genetic alterations in hereditary cancer susceptibility genes in the Ashkenazi Jewish women community of Mexico
Source: Front Genet. 2023 Feb 10;14:1094260. doi: 10.3389/fgene.2023.1094260 (PMC9950094; doi:10.3389/fgene.2023.1094260)
Supplement: Supplementary file 3 [file Table3.docx]

| **ID** | **Gene** | **Region** | **Type of change** | **Transcript** | **Exon** | **cDNA change** | **Protein change** | **Zygosity** |
| --- | --- | --- | --- | --- | --- | --- | --- | --- |
| PJA_305 | *ATM* | exonic | nonsynonymous SNV | NM_000051 | 62 | c.8968G>A | p.Glu2990Lys | Heterozygous |
| PJA_147 | *ATM* | exonic | nonsynonymous SNV | NM_000051 | 52 | c.7778A>G | p.Gln2593Arg | Heterozygous |
| JA56 | *ATM* | exonic | nonsynonymous SNV | NM_000051 | 43 | c.6315G>C | p.Arg2105Ser | Heterozygous |
| PJA_136 | *ATM* | exonic | nonsynonymous SNV | NM_000051 | 43 | c.6315G>C | p.Arg2105Ser | Heterozygous |
| PJA_155 | *ATM* | exonic | nonsynonymous SNV | NM_000051 | 43 | c.6315G>C | p.Arg2105Ser | Heterozygous |
| PJA_228 | *ATM* | exonic | nonsynonymous SNV | NM_000051 | 43 | c.6315G>C | p.Arg2105Ser | Heterozygous |
| PJA_313 | *ATM* | exonic | nonsynonymous SNV | NM_000051 | 43 | c.6315G>C | p.Arg2105Ser | Heterozygous |
| JA54 | *BARD1* | exonic | nonsynonymous SNV | NM_000465 | 6 | c.1568T>C | p.Val523Ala | Heterozygous |
| PJA_154 | *BARD1* | exonic | nonsynonymous SNV | NM_000465 | 6 | c.1568T>C | p.Val523Ala | Heterozygous |
| PJA_271 | *BARD1* | exonic | nonsynonymous SNV | NM_000465 | 6 | c.1568T>C | p.Val523Ala | Heterozygous |
| PJA_239 | *BRCA1* | exonic | nonsynonymous SNV | NM_007294 | 6 | c.400G>A | p.Ala134Thr | Heterozygous |
| PJA_259 | *BRCA2* | exonic | nonsynonymous SNV | NM_000059 | 10 | c.1033A>G | p.Lys345Glu | Heterozygous |
| PJA_104 | *BRCA2* | exonic | nonsynonymous SNV | NM_000059 | 2 | c.62A>G | p.Lys21Arg | Heterozygous |
| PJA_184 | *BRCA2* | exonic | nonsynonymous SNV | NM_000059 | 2 | c.62A>G | p.Lys21Arg | Heterozygous |
| JA04 | *BRIP1* | exonic | nonsynonymous SNV | NM_032043 | 12 | c.1735C>T | p.Arg579Cys | Heterozygous |
| PJA_186 | *BRIP1* | exonic | nonsynonymous SNV | NM_032043 | 19 | c.2706A>G | p.Ile902Met | Heterozygous |
| PJA_228 | *BRIP1* | exonic | nonsynonymous SNV | NM_032043 | 19 | c.2706A>G | p.Ile902Met | Heterozygous |
| PJA_258 | *BRIP1* | exonic | nonsynonymous SNV | NM_032043 | 19 | c.2706A>G | p.Ile902Met | Heterozygous |
| PJA_280 | *BRIP1* | exonic | nonsynonymous SNV | NM_032043 | 19 | c.2706A>G | p.Ile902Met | Heterozygous |
| PJA_313 | *BRIP1* | exonic | nonsynonymous SNV | NM_032043 | 19 | c.2706A>G | p.Ile902Met | Heterozygous |
| PJA_232 | *BRIP1* | exonic | nonsynonymous SNV | NM_032043 | 11 | c.1619A>T | p.Gln540Leu | Heterozygous |
| PJA_253 | *BRIP1* | exonic | nonsynonymous SNV | NM_032043 | 11 | c.1619A>T | p.Gln540Leu | Heterozygous |
| PJA_214 | *CHEK2* | exonic | nonsynonymous SNV | NM_007194 | 2 | c.7C>T | p.Arg3Trp | Heterozygous |
| PJA_234 | *CHEK2* | exonic | nonsynonymous SNV | NM_007194 | 11 | c.1180G>A | p.Glu394Lys | Heterozygous |
| JA03 | *ERCC2* | exonic | nonsynonymous SNV | NM_000400 | 14 | c.1361T>C | p.Val454Ala | Heterozygous |
| JA12 | *ERCC2* | exonic | nonsynonymous SNV | NM_000400 | 22 | c.2068C>T | p.Arg690Trp | Homozygous |
| PJA_110 | *ERCC2* | exonic | nonsynonymous SNV | NM_000400 | 22 | c.2171T>C | p.Met724Thr | Heterozygous |
| PJA_122 | *FAH* | exonic | nonsynonymous SNV | NM_000137 | 5 | c.437A>G | p.Asn146Ser | Heterozygous |
| PJA_123 | *FAH* | exonic | nonsynonymous SNV | NM_000137 | 5 | c.437A>G | p.Asn146Ser | Heterozygous |
| PJA_179 | *FANCI* | exonic | nonsynonymous SNV | NM_001113378 | 4 | c.158G>C | p.Gly53Ala | Heterozygous |
| PJA_189 | *FANCI* | exonic | nonsynonymous SNV | NM_001113378 | 4 | c.158G>C | p.Gly53Ala | Heterozygous |
| PJA_281 | *FANCI* | exonic | nonsynonymous SNV | NM_001113378 | 4 | c.158G>C | p.Gly53Ala | Heterozygous |
| PJA_312 | *FANCI* | exonic | nonsynonymous SNV | NM_001113378 | 4 | c.158G>C | p.Gly53Ala | Heterozygous |
| PJA_327 | *FANCI* | exonic | nonsynonymous SNV | NM_001113378 | 4 | c.158G>C | p.Gly53Ala | Heterozygous |
| JA32 | *KIT* | exonic | nonsynonymous SNV | NM_000222 | 11 | c.1694G>T | p.Gly565Val | Homozygous |
| PJA_113 | *KIT* | exonic | nonsynonymous SNV | NM_000222 | 11 | c.1694G>T | p.Gly565Val | Heterozygous |
| PJA_201 | *KIT* | exonic | nonsynonymous SNV | NM_000222 | 11 | c.1694G>T | p.Gly565Val | Homozygous |
| PJA_205 | *KIT* | exonic | nonsynonymous SNV | NM_000222 | 11 | c.1694G>T | p.Gly565Val | Heterozygous |
| PJA_275 | *MEN1* | exonic | nonsynonymous SNV | NM_000244 | 4 | c.789G>C | p.Gln263His | Heterozygous |
| PJA_255 | *MSH2* | exonic | nonsynonymous SNV | NM_000251 | 16 | c.2714C>T | p.Thr905Ile | Heterozygous |
| JA17 | *MSH2* | exonic | nonsynonymous SNV | NM_000251 | 7 | c.1217G>A | p.Arg406Gln | Heterozygous |
| PJA_304 | *MSH2* | exonic | nonsynonymous SNV | NM_000251 | 3 | c.557A>G | p.Asn186Ser | Heterozygous |
| JA44 | *MSH6* | exonic | nonsynonymous SNV | NM_000179 | 9 | c.3961A>G | p.Arg1321Gly | Heterozygous |
| JA02 | *MSH6* | exonic | nonsynonymous SNV | NM_000179 | 9 | c.3832C>A | p.Pro1278Thr | Heterozygous |
| PJA_150 | *MSH6* | exonic | nonframeshift substitution | NM_000179 | 4 | c.2561_2562delinsTT | p.Lys854_Ile855 | Heterozygous |
| PJA_78 | *MSH6* | exonic | nonsynonymous SNV | NM_000179 | 4 | c.2006T>C | p.Ile669Thr | Heterozygous |
| PJA_146 | *MSH6* | exonic | nonsynonymous SNV | NM_000179 | 4 | c.1061G>A | p.Gly354Glu | Heterozygous |
| PJA_154 | *MSH6* | exonic | nonsynonymous SNV | NM_000179 | 4 | c.1061G>A | p.Gly354Glu | Heterozygous |
| PJA_101 | *NF1* | exonic | nonsynonymous SNV | NM_000267 | 16 | c.1757C>A | p.Thr586Asn | Heterozygous |
| JA40 | *NF2* | exonic | nonsynonymous SNV | NM_000268 | 9 | c.871C>T | p.Arg291Cys | Heterozygous |
| PJA_178 | *PALB2* | exonic | nonsynonymous SNV | NM_024675 | 7 | c.2608G>A | p.Val870Ile | Heterozygous |
| PJA_179 | *PALB2* | exonic | nonsynonymous SNV | NM_024675 | 7 | c.2608G>A | p.Val870Ile | Heterozygous |
| PJA_219 | *PALB2* | exonic | nonsynonymous SNV | NM_024675 | 7 | c.2608G>A | p.Val870Ile | Heterozygous |
| PJA_301 | *PDE11A* | exonic | frameshift insertion | NM_016953 | 20 | c.2763_2764insT | p.Pro922Serfs*27 | Homozygous |
| PJA_143 | *PMS2* | exonic | nonsynonymous SNV | NM_000535 | 2 | c.53T>C | p.Ile18Thr | Heterozygous |
| PJA_145 | *PMS2* | exonic | nonsynonymous SNV | NM_000535 | 2 | c.53T>C | p.Ile18Thr | Heterozygous |
| PJA_245 | *PMS2* | exonic | nonsynonymous SNV | NM_000535 | 2 | c.53T>C | p.Ile18Thr | Heterozygous |
| PJA_209 | *RAD51C* | exonic | nonframeshift deletion | NM_058216 | 9 | c.1125_1127del | p.Glu375_Leu376del | Heterozygous |
| PJA_117 | *RET* | exonic | nonsynonymous SNV | NM_020975 | 14 | c.2544G>A | p.Met848Ile | Heterozygous |
| PJA_336 | *RET* | exonic | nonsynonymous SNV | NM_020975 | 14 | c.2543T>A | p.Met848Lys | Heterozygous |
| PJA_276 | *RET* | exonic | nonsynonymous SNV | NM_020975 | 14 | c.2524G>A | p.Asp842Asn | Heterozygous |
| PJA_277 | *RET* | exonic | nonsynonymous SNV | NM_020975 | 14 | c.2524G>A | p.Asp842Asn | Heterozygous |
