# Supplementary figures and images for: Evaluation of genetic alterations in hereditary cancer susceptibility genes in the Ashkenazi Jewish women community of Mexico
Source: Front Genet. 2023 Feb 10;14:1094260. doi: 10.3389/fgene.2023.1094260 (PMC9950094; doi:10.3389/fgene.2023.1094260)

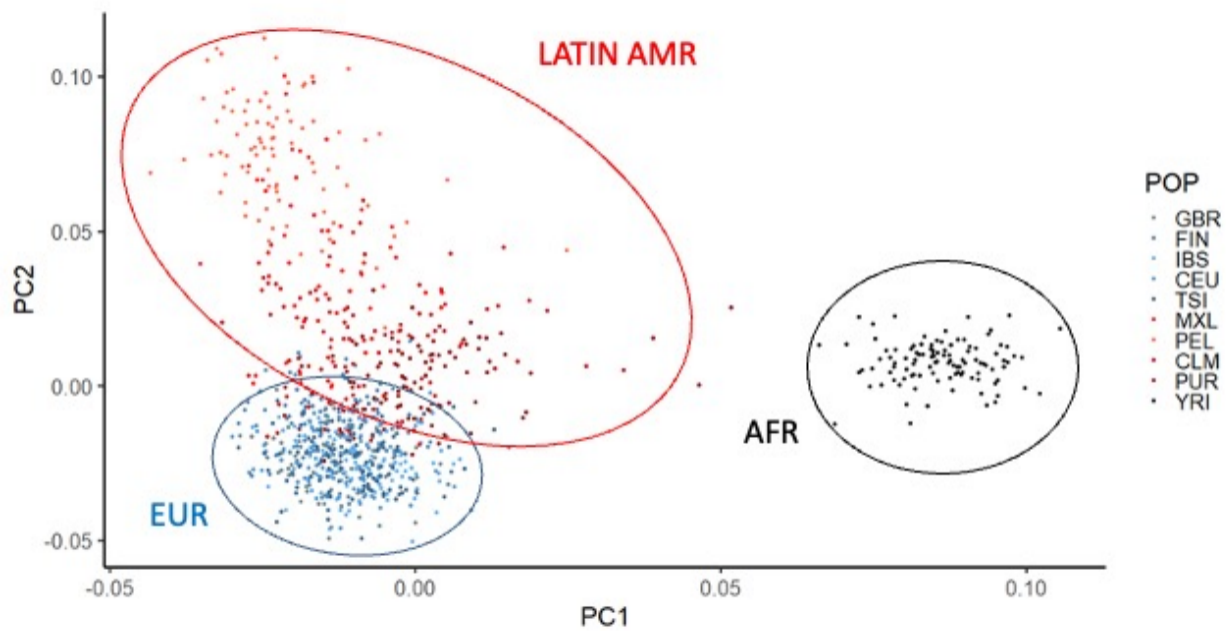

Supplement: Supplementary file 4 [file Image1.pdf]
